# It’s Not Easy Being Agile: Unpacking Paradoxes in Agile Environments

**DOI:** 10.1007/978-3-030-58858-8_19

**Published:** 2020-08-18

**Authors:** Bettina Horlach, Andreas Drechsler

**Affiliations:** 6 IT University of Copenhagen, Copenhagen, Denmark; 7 University of British Columbia, Vancouver, BC Canada; 8 University of Hamburg, Hamburg, Germany; 9 Victoria University of Wellington, Wellington, New Zealand

**Keywords:** Agile teams, Agile organizations, Agile projects, Agile paradoxes

## Abstract

In this paper, we outline inherent tensions in Agile environments, which lead to paradoxes that Agile teams and organizations have to navigate. By taking a critical perspective on Agile frameworks and Agile organizational settings the authors are familiar with, we contribute an initial problematization of paradoxes for the Agile context. For instance, Agile teams face the continuous paradox of ‘doing Agile’ (= following an established Agile way of working) versus ‘being Agile’ (= changing an established Agile way of working). One of the paradoxes that organizations face is whether to start their Agile journey with a directed top-down (and therefore quite un-Agile) ‘big bang’ or to allow an emergent bottom-up transformation (which may be more in-line with the Agile spirit but perhaps not be able to overcome organizational inertia). Future research can draw on our initial problematization as a foundation for subsequent in-depth investigations of these Agile paradoxes. Agile teams and organizations can draw on our initial problematization of Agile paradoxes to inform their learning and change processes.

## Introduction

Agile and hybrid project environments are increasingly becoming the norm within and even beyond the IT industry, and organizations increasingly start scaling Agile^1^ beyond IT project teams [1]. There are numerous methodologies for Agile project management and scaling Agile, which claim to embody the Agile Manifesto’s principles and values (e.g., Scrum, SAFe, Disciplined Agile etc.). Studies show that embracing Agile leads to generally satisfied individuals and companies, but there are also a variety of obstacles that teams and organizations may face [2–4].

In a more general perspective, “most management practices create their own nemesis” [5 p. 491], and Agile is no exception. As one role of research is to critique the status quo [6], we do so in this paper for the Agile context by outlining areas of tension which result in paradoxes that Agile teams and organizations running Agile teams may have to navigate. Following Putnam et al. [7], we define paradoxes as “contradictions that persist over time, impose and reflect back on each other, and develop into seemingly irrational or absurd situations because their continuity creates situations in which options appear mutually exclusive, making choices among them difficult” (p. 72).

By providing this critique, we problematize [8] Agile beyond a functionalist view that is centered on performance or effectiveness. Our initial problematization therefore paves the way for future, more in-depth research contributions that investigate each paradox – as an instance of ‘the dark side of Agile’ – more closely. We see these paradoxes as a starting point for more focused theoretical and empirical investigations how Agile teams and organizations encounter, experience, and cope with these Agile paradoxes. As one key tenet of Agile organizations is continuous learning and change, such in-depth treatments of Agile paradoxes can therefore also contribute to organizational learning and change efforts in practice.

Our analysis draws on a critical reading of selected Agile methodologies and techniques, the Agile research literature, as well as a critical assessment of Agile environments that the authors are familiar with (see Sect. 2). Note that while there is a quite comprehensive dataset that informed the authors’ other research in Agile organizational contexts, there was no specific data analysis conducted for this paper to inform our initial problematization of Agile paradoxes. We see such an undertaking as a fruitful endeavor for future research.

In this short paper, we first outline the backdrop against which we provide our critique. We then start discussing sources for agile paradoxes on the levels of the Agile team as well as on the organizational level for those organizations who scaled Agile beyond individual teams.

## Empirical Background

Both authors are involved in a large-scale cross-industry and cross-country research program on Agile organizational transformation and have collected extensive data across two phases. The first data collection phase consisted of interviews and focus groups with seven executives (e.g., CIO or CDO), whereas the second phase consisted of interviews with lower level managers (e.g. program managers, product owners, enterprise architect) or external consultants. The participants had two essential criteria to fulfill: 1) their organization is undergoing a transformation towards organizational agility, and 2) the participants hold a position with in-depth insights on the overall (agile) organizational system. For the executives group, we conducted three single day focus group workshops [9] and seven semi-structured interviews. For the other group, we conducted 33 semi-structured interviews. Each interview session lasted 45–75 min and was audio-recorded and transcribed.

All gathered data has been qualitatively analyzed to inform research on the implications of Agile for topics such as portfolio management [10], enterprise architecture [11], business/IT alignment [12], and IT governance (currently under review). Beyond these specific topics, however, the authors also observed more general patterns of a paradoxical nature in the Agile organizational contexts the interview and focus group participants gave insight into, and likewise in the (Scaling) Agile frameworks that the interviewees referred to. The following two sections outline the sources for these paradoxes that the authors have identified. Due to space restrictions in this short paper, we are only able to outline and problematize each paradox on a rather general level.

## Sources for Agile Paradoxes on the Team Level

### Being Agile Versus Doing Agile

The different aspects of Agile such as values, principles, methodologies, or techniques allow us to distinguish between teams that are ‘being Agile’ (i.e., embrace Agile principles and values in an Agile mind-set and truly focus on delivering customer value while learning continuously) and ‘doing Agile’ (i.e. adopt an Agile methodology or a set of Agile techniques and simply follow them). Note that ‘doing Agile’ can be a step on the way of towards fully embracing the Agile mindset [13, 14]. However, there is the danger that an Agile team stops advancing beyond the ‘doing Agile’ stage, i.e. it keeps trying to ‘perfect’ their adoption of their chosen Agile approach. In contrast, teams ‘being Agile’ commit themselves to being accountable for their work, being willing and able to handle uncertainty in their work, and to strive for continuous improvement. The specific way of working (methodology, process, techniques, tools) or any form of adherence is less important. In this sense, the term ‘Agile methodology’ is already paradoxical in itself, as the term ‘methodology’ implies a specific prescription. Especially in volatile environments or in environments where an Agile methodology or framework forms the cornerstone of the Agile transformation, there may be a permanent paradoxical tension between ‘doing Agile’ and ‘being Agile’ for Agile teams.

### Experience Versus ‘Appetite’ for Change and Flexibility

Agile environments are built around the assumption that information completeness is never achieved due to ever-changing environments and customer needs. Hence, a high level of readiness for coping with change is a critical factor for Agile team effectiveness. However, a high amount of team members’ experience in particular may also be a source for a paradox. A team member’s experience can come from traditional project environments (particularly since Agile is still a quite young trend) and therefore include a preference for stable processes and predefined requirements based on detailed planning. Each Agile team member also continuously gains experience in (and may become accustomed to) their particular Agile approach and also regarding the artefact they are working on. Both variants of experience are challenged, however, by Agile’s ‘permanent uncertainty’ in its mindset. Sometimes, a radical change to the way of working or the deliverable may be what the situation or the market requires, and extant experiences may be source for resistance or inertia regarding those changes. The paradox here is therefore that an increase in individual and collective experience may lead to a decreased ‘appetite’ for future change and therefore to less flexibility for a team.

### Exploration Versus Exploitation

Agile teams are also characterized by a high level of self-organization and decision-making autonomy. In traditional Agile teams, this autonomy mainly concerns the choice of and ongoing changes to the methodology, techniques, and tools [15–17]. In Scaling Agile teams, this autonomy often extends to product or service design changes and future directions for their product(s)/service(s)/area(s) [18, 19]. In the former case, a paradox arises out of the tension between the requirements of getting work done and continuously sharpening (and potentially re-learning) one’s – metaphorical and literal – tools. This may pose the danger of splitting a group into those advocating change and those advocating getting things done. Autonomy over one’s artefact in the latter case could lead to a similar paradoxical scenario of the well-researched tension between exploration vs. exploitation [20, 21]. Should a team radically re-invent the artefact to adapt to or anticipate market changes, or incrementally refine the artefact to fine-tune it to established customer needs? In both cases, the team’s handling of this paradox would enable or constrain future actions.

### Directed Versus Emergent Team Process Change

As the notion of continuous change is built into Agile environments and teams, roles such as the Scrum Masters and Agile coaches are responsible for guiding and supporting the Agile team towards becoming more effective. However, there are two general archetypes how these roles could be set up (or choose by themselves) to fulfill their task: Agile coaches and Scrum Masters could either direct a team’s development according to what they perceive as best for the team (to be an ‘Agile leader’ or even an ‘Agile police’, so to speak), or could nurture the teams instead (i.e. ‘help the people to help themselves’) and let any changes to a team’s way of working emerge from within the team. In the former case, having change directed and induced from outside the team could potentially undermine a team’s autonomy. On the flip side, a team that is perhaps ‘too comfortable’ with their current Agile approach may not engage in a self-transformation without external direction even though it would benefit from certain changes [22]. Either way, Scrum masters and Agile coaches could even oppose or counteract good Agile practices – perhaps just subconsciously – in order to continuously create their own work in order to be kept employed or contracted and make themselves seemingly indispensable. The underlying paradox here is the one of balancing team autonomy with external directions with respect to changes to the team’s way of working.

## Sources for Agile Paradoxes on the Organization Level

### Starting/Realizing the Agile (Self-)transformation: ‘Big Bang’ Versus Emergence

When aiming to introduce Agile on a larger scale, organizations have to choose an approach that lies somewhere between an initial ‘big bang’ top-down transformation towards Agile or an incremental, iterative, and emergent approach where different parts of the organizations can choose whether and how they adopt Agile [23, 24]. In other words, how Agile should the Agile transformation itself be, and how much predefined structures and processes should the first target state have? For instance, in one situation a common way of working across several Agile teams or units may be more effective to successfully transform (parts of) the organization, whereas in another situation self-taught bottom-up experimentation with Agile techniques and tools may be the more effective approach – particularly when considering how to set the stage for ‘being Agile’ in a longer-term and sustained perspective. As Agile implies a high degree of team autonomy instead of having top-down pre-planned decisions, a ‘big bang Agile introduction’ is therefore paradoxical in itself. The danger of mixed messages during an Agile transformation lies in a regression to a directive (i.e. non-autonomous) way of working and organizational culture, and also would constrain the Agile units’ autonomy to self-transform in the future. Simultaneously, unfettered team autonomy right from the start could lead to the danger of an aimless or quickly stalling transformation process.

### Directing Teams Versus Team Autonomy

The tension between directing and simultaneously sustaining autonomous Agile teams may not only occur during the initial Agile transformation but may stay with organizations throughout their entire Agile journey. The nature of the resulting paradox, however, shifts to issues related to focus, resources, effectiveness, and efficiency. The *focus* component affects how a team’s strategic direction is set and influenced. While each team may know their product’s customers best, an organization’s top management may wish to change or retire some products. In this situation, the tension arises whether a team should be in charge of a changed purpose or even its own dissolution, or whether an organization wants to override its teams’ autonomy in these cases. With respect to *staffing and resourcing*, the Agile idea generally implies that a team would be responsible for the resources they require to fulfill their purpose. However, resource scarcity in organizations, competition for resources across teams, and the willingness to achieve a global optimum across teams may prevent a purely bottom-up decision-making on resources. Again, the organization would paradoxically interfere with a team’s autonomy if it denies requested necessary resources. Measuring Agile team *effectiveness or performance* is another source for paradoxes. Measuring performance could have the purpose of identifying the extent to which an Agile team contributes business value, or the purpose of aligning teams with overarching strategic objectives. In both cases an organization would again interfere directly with team autonomy. Finally, *efficiency* concerns the way of working throughout the organization, i.e. should teams be provided with or even have to adhere to a common set of Agile values, processes, techniques, and tools, which would allow team members to be shared or move between teams without having to adjust to fundamentally new ways of working? On the other hand, an organization-wide ‘Agile standard’ is again a paradox in itself, since one emphasis of Agile lies on continuous change and adaptability, and different Agile approaches may be effective for different teams. All these aspects are manifestations of a systemic contradiction of having autonomous teams within a coherent business organization. In a nutshell, any decision above the team level may ultimately undermine the teams’ perceived autonomy.

### Team Identity and Purpose Versus the Need for Radical Business Change

When an Agile team in a Scaled Agile environment is made responsible for (a) particular product(s)/service(s)/area(s), it achieves its sustained focus through this purpose. Over time, having a consistent focus and purpose contributes to a team’s shared identity. However, being responsible for a specific product or service for quite a long period may lead to a ‘blindness’ and attachment of teams to their built artefact. Consequently, a team may add unnecessary bells and whistles to ‘their’ artefact to justify the product’s as well as the team’s continued existence and resourcing in comparison to other teams. A team may also become protective of ‘their’ product or service (area) instead of recognizing the need for a radical change or its retirement, in order to fulfill and surpass changed customer needs and support the organization in thriving in the changing business environment. A team’s purpose may therefore become a self-referential part of its identity so that strong repressions of or reactions against a radical change to the purpose occur, with the unanticipated consequence of limiting the effective team agility to self-transform when necessary. The paradox here therefore is that the same mechanisms that keep an Agile team together and effective may also hinder its ability to detect the best time and ways to re-invent themselves for their best possible contribution to organizational value.

## Discussion, Conclusion, Outlook

In this paper, we identified and briefly discussed several potential paradoxes in Agile contexts. Through our discussion of these Agile paradoxes, we contribute a problematization [8] of Agile on a deeper level than a functionalist perspective that analyzes ‘what works’ [25], a critique of Agile as a management fashion [26], or previous attempts at identifying Agile paradoxes [27]. In our problematization, we interrogated key Agile tenets and found that embracing Agile may produce a number of paradoxes on the team and the organizational level. We do not see these paradoxes’ existence as a negative thing. In fact, to harness the true potential of Agile transformations organizations may need to become adept at continuously confronting these paradoxes and utilizing their forces in a constructive and not a destructive way for their ongoing self-transformation. Since learning and change are two key Agile tenets, Agile organizations may be uniquely positioned to incorporate the confrontation with their paradoxes into their ‘business as usual’, instead of treating tensions and paradoxes as issues that stand in the way of organizational effectiveness and need to be resolved. While we have not investigated each paradox in-depth, our initial problematization may still be useful to guide and inspire [28] learning and change processes in Agile organizations.

Some of the underlying tensions – such as the exploration vs. exploitation one – are already well known in the literature [20, 29]. Others – such as the tensions around Agile team autonomy – may be specific for Agile environments and transformations. They have – to the authors’ best knowledge – not been thoroughly investigated yet. Our problematization therefore contributes to a comprehensive research agenda to investigate how Agile teams and organizations encounter, experience, and cope with paradoxes on their Agile journeys. We therefore encourage empirical validation and extension of our findings, as the paradoxes in this paper are limited by being based on general insights from IT organizational roles within two countries. Thus, we also advocate for analyzing tensions perceived by the business side in order to capture a truly comprehensive perspective on the paradoxes.

## Footnotes

1 For brevity, we use the term Agile (with a capital A) in this paper as a term encompassing agile values, principles, methodologies, and techniques, without referring to specific ones.
